# A new type of elastic fixation, using an encircling and binding technique, for tibiofibular syndesmosis stabilization: comparison to traditional cortical screw fixation

**DOI:** 10.1186/s13018-023-03579-x

**Published:** 2023-04-03

**Authors:** Nan Zhu, Qigang Zhong, Junfeng Zhan, Shuo Zhang, Wei Liu, Yunfeng Yao, Juehua Jing

**Affiliations:** Department of Orthopedics, Second Affiliated Hospital of Anhui Medical University, Hefei, China

**Keywords:** Ankle joint, Elastic fixation, Syndesmosis, Physiological micromotion

## Abstract

**Background:**

The distal tibiofibular syndesmosis (DTS) is a complex fibrous joint that contributes to the stability and weight-bearing function of the ankle. As such, repair of DTS injury is required, providing fixation strength while maintaining ankle range of motion. The aim of this study was to compare a new elastic fixation technique, using an encircling and binding technique, for DTS stabilization, compared to the traditional cortical bone screw fixation.

**Methods:**

This was a retrospective analysis of 67 patients treated for a DTS injury at our hospital, between June 2019 and June 2021. Of them, 33 were treated with encircling and binding (EB group) and 34 using a cortical screw (CS group). The following outcomes were compared between groups: time to inferior tibiofibular fixation; length of hospital stay; time to partial weight bearing; time to complete weight bearing; complications; imaging data; and functional scores.

**Results:**

Successful stabilization was achieved in all cases, with a mean follow-up period of 15.78 ± 2.97 months. Time to fixation and time to partial and complete weight bearing were shorter for the EB than that for the CS group. The length of hospital was not different between groups. With regard to complications, a superficial infection developed in one patient in each group, with wound healing achieved after active treatment. Screw fracture occurred in two patients in the CS group. At 3 months post-surgery, the American Foot Surgery Association Ankle-Hindfoot score (AOFAS) was higher and the pain score lower for the EB than that for the CS group, but with no between-group difference at the final follow-up. On imaging, the tibiofibular clear space and tibiofibular overlap were not different between groups.

**Conclusions:**

DTS fixation using encircling and binding yielded better clinical and functional outcomes than did cortical screw fixation at 3 months post-surgery, with no difference at the final follow-up. This novel fixation technique provides firm fixation, combined with earlier return to postoperative exercise and recovery of ankle function.

## Background

The stability of the distal tibiofibular syndesmosis (DTS) complex, which contributes to the stability and load-bearing capacity of the ankle joint, is provided by the anterior inferior tibiofibular, posterior inferior tibiofibular, inferior transverse, and interosseous ligaments [1]. Injury to any two of these ligaments will result in ankle joint instability, with a change in the contact area of the tibiotalar joint surface. Dattani et al. [2] demonstrated that a 1-mm displacement of the talus within the ankle mortise can reduce the tibiotalar contact area by 42%, aggravate articular cartilage wear, and lead to complications, such as chronic pain, traumatic arthritis, and ankle instability. Anatomical reduction and rigid internal fixation for a DTS injury is thus required.

Fixation using a cortical screw has been considered the gold standard for treatment of a DTS injury, providing a local stable environment, with restraint against external rotational stress, to allow remodeling of the ligaments [3]. However, as the DTS is a fibrous joint, with a range of motion (ROM) of 2–5° along all three planes of motion, coronal, sagittal, and transverse [4], the screw must be removed before weight bearing to avoid metal fatigue, which can lead to screw fracture. This complication can increase the risk of perioperative infection, functional ankle joint impairments, and the economic burden to patients. Moreover, owing to the hardness of cortical screws, any deviation in the placement of the screw can result in misalignment of the fibula in the fibular notch and in ankle stiffness. This is an important clinical issue considering that the incidence of poor reduction of the DTS is high, at 25.5–52% for medial collateral ligament (MCL) injuries [5]. As such, the use of rigid fixation for DTS injuries has gradually been replaced by elastic fixation to retain a certain ROM while satisfying the requirement for strength of the fixation and to avoid the related complications caused by long-term rigid fixation [6, 7].

Different types of elastic fixation techniques have been described, including autogenous ligament repair, artificial ligament repair, rivet technique, endo-button system, and suture-button system [8–10]. Among these, the suture-button system has been associated with good imaging outcomes and ankle function scores on follow-up; however, complications, such as sinking of the internal fixation device, osteolysis, and enlargement of tibial boreholes, have been reported [8, 11]. The above has highlighted the importance of the DTS for ankle stability and the need for anatomic reduction and strong internal fixation for surgical treatment. Currently, the commonly used fixation methods have their own advantages and disadvantages. How to have a fixation method that can not only achieve the fixed strength of rigid fixation but also satisfy the elastic fretting of the joint under the premise of few complications is still a hot topic in the research of foot and ankle surgery.

The strength of the elastic fixation can be improved by using a nice knot, which is a double-layer folding and sliding knot that provides high tension and stable fixation, which is maintained over time, while resisting fatigue of the fixation and retaining the ROM characteristics of the DTS joint [12–14]. Based on the positive outcomes reported for the nice knot as fixation for other fractures, we used the new nice knot encircling technique, via a superior tunnel approach, to provide elastic fixation of the DTS after injury. Our aim in this study was to compare the clinical and functional outcomes of our novel elastic DTS fixation technique with that achieved by the traditional use of cortical screws.

## Methods

### Patients

This was a retrospective study of patients treated for a DTS injury at our hospital, between June 2019 and June 2021. The methods of our study were approved by our Hospital Ethics Committee, and all patients provided informed consent for use of their data for research and for publication. All treatment options are based on informed consent of the patient. The patients were assessed as follows: inclusion criteria—a DTS gap > 6 mm or overlap < 6 mm; age between 18 and 65 years; a positive Hook’s test and external rotation stress test intraoperatively, before reduction; and complete data for a follow-up ≥ 1 year; exclusion criteria—open or chronic fracture; severe osteoporosis; and prior ankle joint dysfunction, such as traumatic arthritis, congenital ankle deformity, or Kashin–Beck disease.

### Surgical procedure

All patients were initially managed with immobilization and elevation until the status of the soft tissues was judged to be conducive to safe surgical treatment. Patients were informed of surgical risks and complications before surgery. When the DTS was to be fixed intraoperatively, conventional screw fixation was not conducive to early postoperative functional exercise, and a second operation was required to remove the screw, and complications such as internal fixation fracture, loosening may occur easily. All surgeries were performed by the same group of surgeons and under epidural anesthesia. Patients were placed in the supine position and a tourniquet applied. Lateral malleolus fractures were treated with anatomical reduction and rigid internal fixation with plates and screws. Internal reduction and fixation, via a posterolateral approach, were used for lateral and posterior malleolar fractures. Medial malleolar fractures were treated with internal reduction and fixation using a hollow screw or Kirschner wire via an open medial approach. Triangular ligament injuries were reconstructed using thread rivets. After fracture fixation and ligament reconstruction, Hook’s test and the lateral rotation test were then performed under C-arm fluoroscopy to evaluate the degree of DTS injury.

### Cortical screw fixation of the DTS

After the normal tibiofibular space was restored, a bone tunnel was created, 3.0 cm above the articular surface of the distal tibia, from the posterolateral to the anteromedial side of the tibia through the three layers of the bone cortex, using a 2.5-mm drill bit. The 3.5-mm full-threaded cortical screws were then implanted for fixation of the DTS.

### Encircling and binding fixation of the DTS

**Fig. 1:**
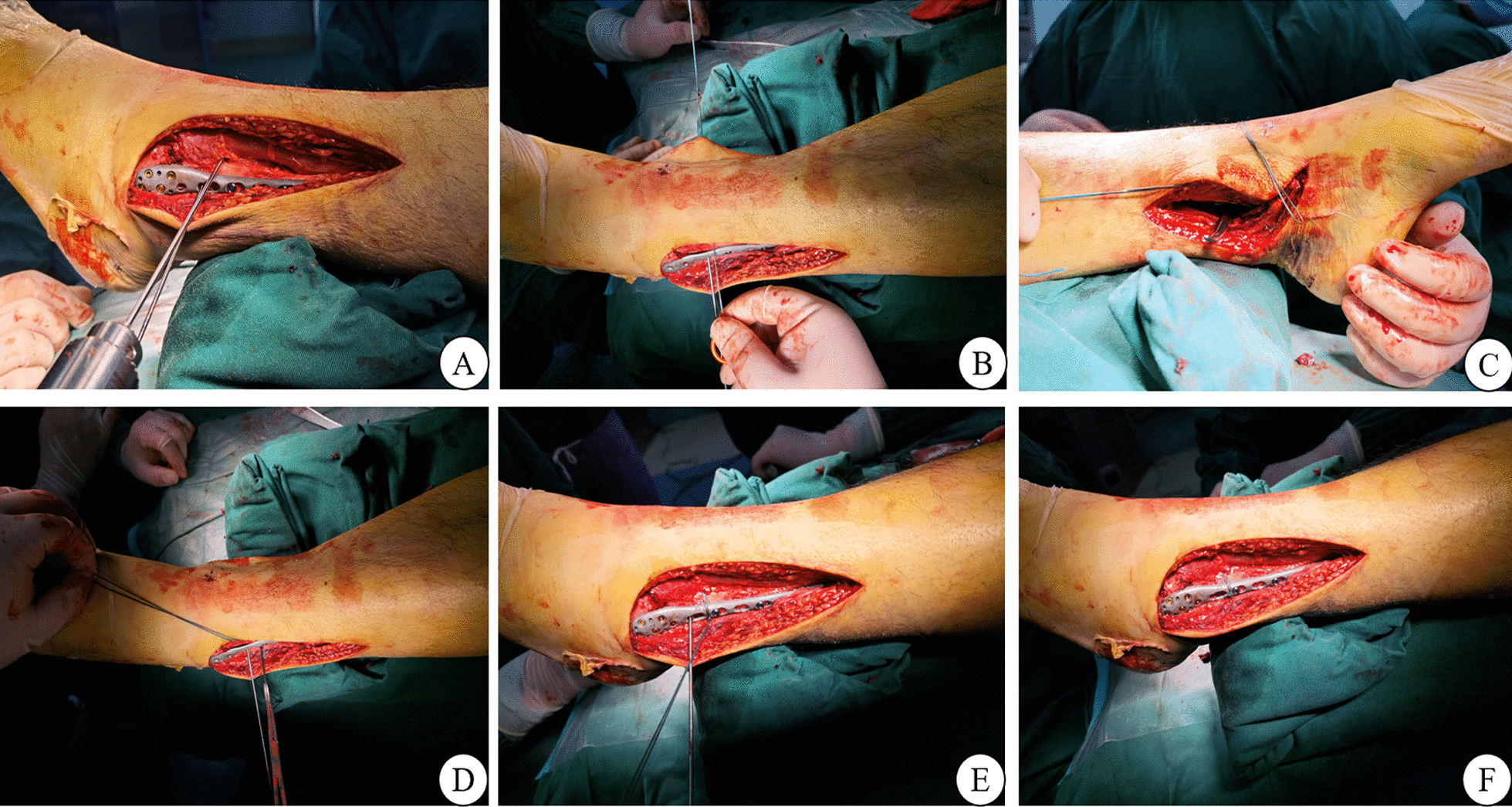
Detailed steps of the intraoperative fixation of the distal tibiofibular syndesmosis using the encircling and binding technique. **A** A 2.5-mm Kirschner wire placed on the anterior edge of the fibula is used to create a bone canal. **B** Use of a threading device to introduce double-strand sutures from the inside to the outside through the bone canal. **C**, **D** Vascular clamp blunt separation, close to the posterior edge of the tibia, clamping the medial double strands and pulling back to the outside. **E** Making the nice knot on the posterior edge of the fibular plate. **F** After obtaining satisfactory tension, 3–4 single-wire knots fixed to the nice knot form an encirclement

**Fig. 2:**
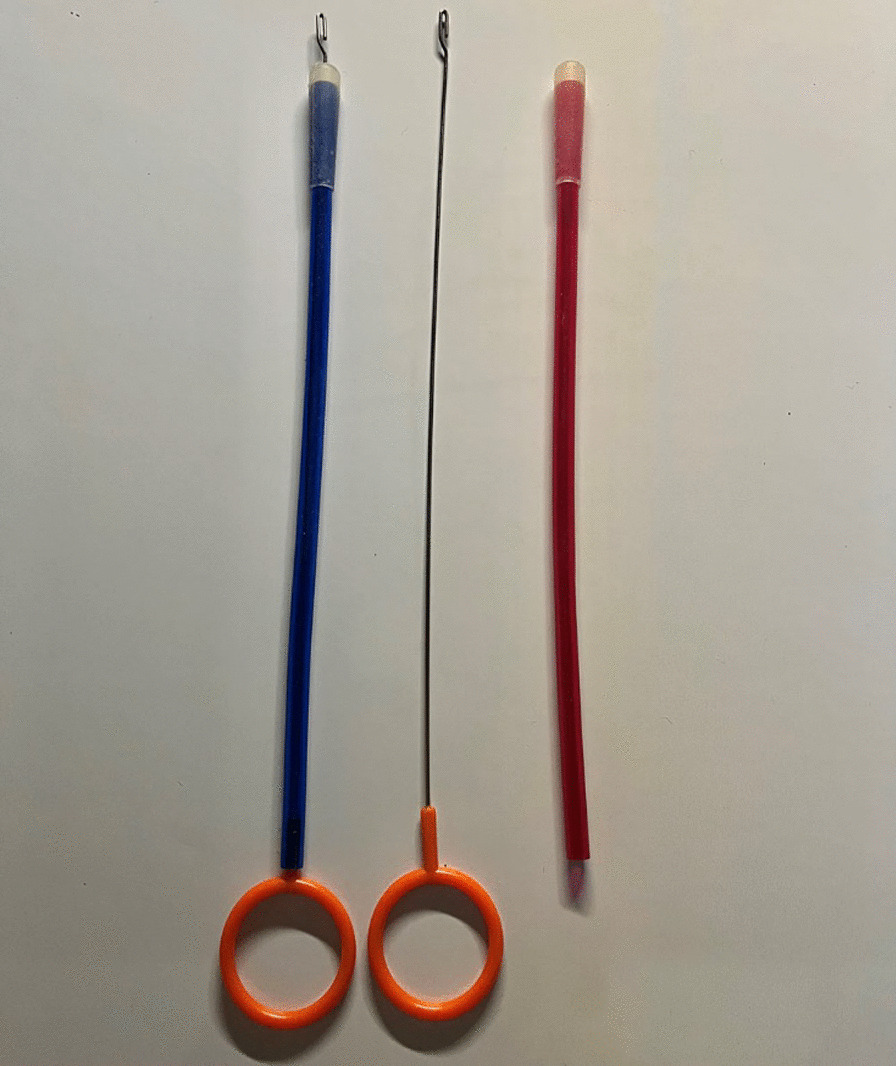
Tools for wire crossover used to introduce the double strands of high-strength polyester, non-absorbable, suture through the bone canal

The detailed steps for the EB technique are shown in Fig. 1. With the ankle joint placed in 5° of dorsiflexion, the DTS was reduced with temporary point fixation performed with forceps. The normal tibiofibular space, located 3.0 cm superior to the articular surface of the distal tibia, was reduced. A bone tunnel was then created using a 2.5-mm-diameter Kirschner wire, localized at 25–30° in the coronal plane and 10–15° in the horizontal plane. Tools for wire crossover are shown in Fig. 2 and were used to introduce double strands of high-strength polyester, non-absorbable, suture through the bone canal, from the outside to the inside. The medial suture was then pulled back to the lateral side, close to the posterior end of the tibia, using vascular forceps, and a nice knot made on the lateral side of the fibula. After adjusting to the appropriate tension, the nice knot was locked using 3–4 single knots and the reduction forceps were loosened and removed. Once the fixation technique was completed, the stability was verified under C-arm fluoroscopy and the incision was washed and sutured.

### Postoperative management

After surgery, a cotton gauze bandage was applied with an elastic wrap, and the limb was elevated. Routine anti-inflammation, detumescence, and pain relief were provided. At 24 h postoperatively, active ankle ROM in flexion/extension was initiated, with a gradual increase in exercise intensity. Weight bearing was initiated at 8 weeks postoperatively, progressing to full weight bearing as tolerated.

### Evaluation index

The following outcomes were evaluated: intraoperative time of fixation of the DTS; operative time; length of hospital stay; time to partial weight bearing; and time to full weight bearing. During the follow-up period, complications such as incision infection, poor wound healing, deep venous thrombosis, loosening of the elastic fixation device, fracture of the inferior tibiofibular screw, and loss of reduction were closely monitored. Patient-reported outcomes included the visual analog scale (VAS) for pain and the American Foot Surgery Association Ankle-Hindfoot Score (AOFAS) for pain, function, and objective measurement of alignment. The reduction criteria were evaluated according to the measurement of the tibiofibular space (TFCS) and inferior tibiofibular overlap distance (TFOS) on radiographs obtained immediately after surgery and at the last follow-up.

### Statistical analysis

Data were reported as the mean and standard deviation. Between-group differences were evaluated using an independent sample *t*-test for continuous data and the Chi-squared (*X*^2^) or Fisher’s exact test for categorical data. Within-group comparison between time points was evaluated using paired-sample *t*-test or one-way analysis of variance for normally distributed data and a rank sum test for non-normally distributed data. A *p* value < 0.05 was significant. All analyses were performed using SPSS (version 26.0).

## Results

There were 312 ankle fractures identified during the study period, 94 presented with a DTS injury, with 67 meeting our inclusion criteria. Patients were divided into two groups accordingly for analysis based on the intraoperative DTS treatment: cortical screw (CS, *n* = 34) and elastic encircling and binding (EB, *n* = 33) groups. The distribution of ankle injuries involving the DTS for both groups was as follows. The CS group included sprains (*n* = 6), traffic injury (*n* = 16), fall from a height (*n* = 9), and direct trauma (*n* = 3). The EB group included sprains (*n* = 5), traffic injury (*n* = 16), fall from a height (*n* = 8), and direct trauma (*n* = 4). The types of injuries, according to the Lauge–Hansen classification [15], were as follows for the CS and EB group, respectively: pronation-external rotation, 12 and 13 cases; pronation–abduction, 17 and 16 cases; and supination-external rotation, 5 and 4 cases. The general characteristics for the two groups are given in Table 1. Successful fixation was achieved after DTS reduction in all cases. The perioperative and follow-up data are presented in Table 2, with a shorter operative time, time to partial weight bearing, and time to full weight bearing observed for the EB group compared to those for the CS group (*p* ≤ 0.023).

**Table 1:** Comparison of general data between the cortical screw (CS) and encircling and binding (EB) groups

Patients	EB group (*n* = 33)	CS group (*n* = 34)	Between-group difference Independent group *t*-test (*t*) or Chi-squared (*X*^*2*^)	*p* value
Age (years)	34.36 ± 14.43	39.76 ± 14.50	*t*, − 1.528	0.131
Sex (*n*, F/M)	23/10	24/10	*X*^2^, 0.006	0.936
Side (*n*, L/R)	17/14	15/19	*X*^2^, 0.746	0.388
BMI (kg/m^2^)	25.54 ± 4.15	25.30 ± 4.02	*t*, 0.240	0.811
Smoking history (*n*)	23/10	23/11	*X*^2^, 0.033	0.856

*BMI* Body Mass Index, *F* female, *M* male, *L* left, *R* right, *CS* cortical screw, *EB* encircling and binding

**Table 2:** Comparison of perioperative data between the encircling and binding (EB) and cortical screw (CS) groups

Perioperative data	EB group (*n* = 33)	CS group (*n* = 34)	Paired *t*-test	*p* value
Fixation time (min)	12.58 ± 2.01	16.44 ± 2.34	− 7.238	**0.000**
Operative time (min)	116.12 ± 29.78	118.62 ± 31.50	− 0.333	0.740
Duration of hospitalization (days)	15.03 ± 4.37	16.82 ± 5.84	− 1.420	0.160
Time to partial weight bearing (weeks)	5.75 ± 1.05	6.62 ± 1.16	− 3.228	**0.002**
Time to complete weight bearing (weeks)	10.01 ± 1.82	11.07 ± 1.90	− 2.321	**0.023**

The bold words in the chart indicate statistical significance

*CS* cortical screw, *EB* encircling and binding, *Fixation time* the time of fixation of the lower tibiofibula

All patients were followed up for > 1 year, with an average follow-up period of 15.78 ± 2.97 months. The follow-up data for both groups are given in Table 3. Within-group improvement, from immediate postoperative to the final follow-up, in VAS and AOFAS was identified for both groups (*p* = 0.00), but with no significant change in the TFOS and TFCS. Between-group differences were as follows. At 3 months postoperatively, the VAS was lower and the AOFAS higher for the EB group than for the CS group, with no between-group difference at the final follow-up, although the AOFAS remained slightly higher for the EB group and the VAS slightly lower for the CS group.

**Table 3:** Comparison of follow-up data between the two groups of patients

Follow-up data	Time	EB group (*n* = 33)	CS group (*n* = 34)	Independent group *t*-value	*P* value
VAS	Immediately post	5.82 ± 1.67	6.06 ± 2.10	− 0.518	0.606
	3 months post	2.09 ± 1.18	3.32 ± 1.34	− 3.985	**0.000**
	Last follow-up	0.94 ± 0.75	1.00 ± 0.82	− 0.317	0.753
*F* value		134.528	91.079		
*P* value		**0.000**	**0.000**		
AOFAS	Immediately post	41.21 ± 8.04	41.41 ± 7.44	− 0.106	0.916
	3 months post	78.09 ± 9.11	68.50 ± 8.89	4.360	**0.000**
	Last follow-up	95.33 ± 5.70	92.70 ± 5.69	1.888	0.064
*F* value		525.355	422.517		
*P* value		**0.000**	**0.000**		
TFCS	Immediately post	3.87 ± 0.55	3.93 ± 0.53	− 0.443	0.660
	3 months post	3.96 ± 0.76	3.83 ± 0.63	0.772	0.443
	Last follow-up	3.95 ± 0.75	3.88 ± 0.62	0.393	0.696
*F* value		0.147	0.230		
*P* value		0.865	0.796		
TFOS	Immediately post	7.97 ± 1.18	8.30 ± 1.06	− 1.198	0.235
	3 months post	8.25 ± 0.97	8.28 ± 0.97	− 0.108	0.914
	Last follow-up	8.22 ± 1.18	8.21 ± 1.19	0.067	0.947
*F* value		0.726	0.095		
*P* value		0.488	0.909		

The bold words in the chart indicate statistical significance

*CS* cortical screw, *EB* encircling and binding, *VAS* visual analog pain score, *AOFAS* American Foot Surgery Association Ankle-Hindfoot Score, *TFCS* tibiofibular space, *TFOS* tibiofibular overlap distance

With regard to complications, there was one case of superficial wound infection in each group, with healing achieved in one stage in both cases with regular antibiotic and anti-inflammatory treatment, dressing change, local phototherapy in physical therapy, and rest. There was no incidence of deep infection, deep venous thrombosis, or pulmonary embolism.

**Fig. 3:**
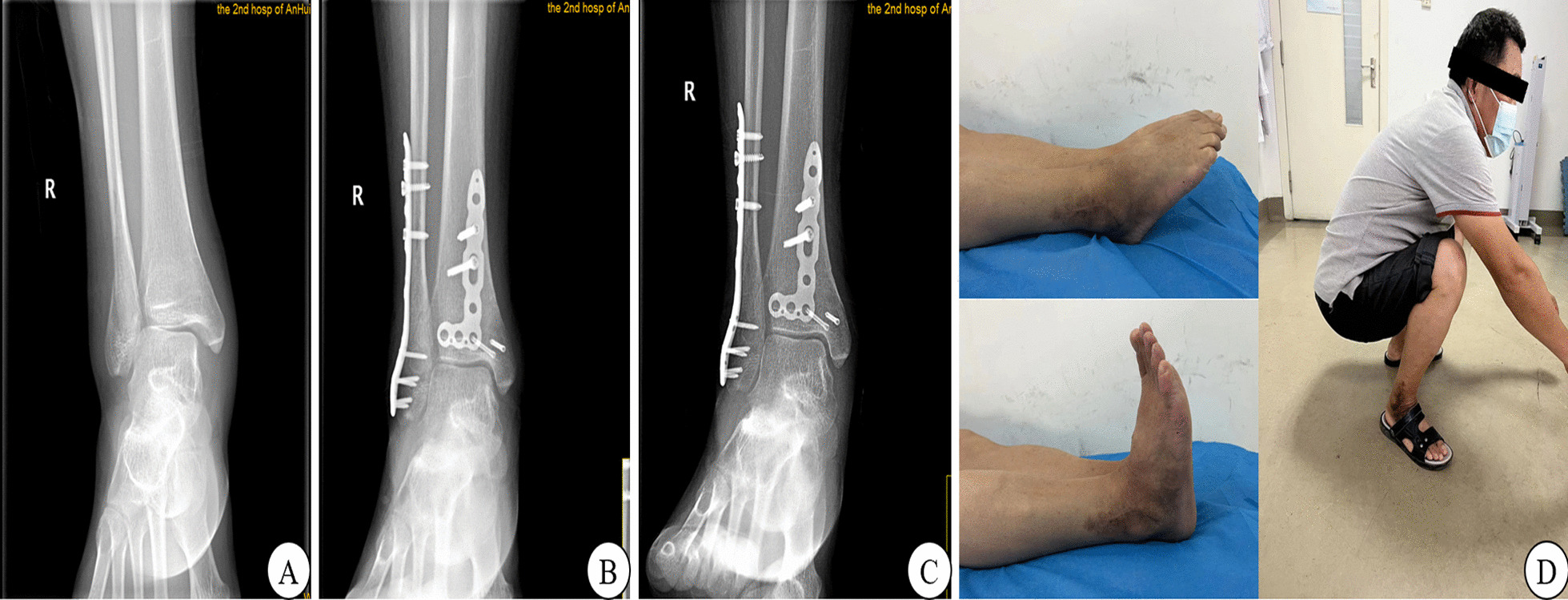
A typical case of distal tibiofibular syndesmosis injury treated with the encircling and binding technique. **A** Preoperative radiograph shows a fracture of the right ankle with injury to the distal tibiofibular syndesmosis. **B** Three months after surgery, radiography reveals that the fracture has healed well and there is no loss of reduction of the distal tibiofibular syndesmosis. **C** One year after surgery, radiography shows that the internal fixation is in place and there is no loss of reduction. **D** Ankle function at the last follow-up

Implants, including plates and screws, were removed after fracture healing in both groups. The inferior tibiofibular screw was removed 2–3 months after internal fixation in the CS group. In this group, screw fracture occurred in two cases. The fixation line of the DTS was routinely removed for those in the EB group. A representative case of encircling and binding is shown in Fig. 3. After surgery and up to the last follow-up, the internal fixation for the EB group remained firm, with no loosening of the internal fixation device or loss of reduction.

## Discussion

Our findings demonstrate the feasibility of an elastic fixation, using an encircling and binding technique, to provide firm stability of the DTS after injury. This is an important finding considering the importance of the DTS to ankle stability and its involvement in common ankle injuries, including, according to the Lauge-Hansen classification [4], pronation–abduction types II and III, pronation-external rotational type IV, and supination-external rotation injuries. Van Heest and Lafferty [16] also reported a DTS injury in approximately 5% of MCL injuries, 10% of ankle sprains, and 23% of ankle fractures. The complexity of ankle motion, which includes flexion/extension along the sagittal plane, talus rotation, and fibular translation, underlines the importance of achieving and maintaining DTS stability for normal ankle joint function.

Lateral fixation of the DTS using cortical screws is the traditional treatment for DTS injuries; however, the use of cortical screws is associated with complications, such as poor reduction, screw fracture and loss of reduction requiring secondary surgery, and decrease or loss of the tibiofibular clear space [17–19]. Moreover, there remains controversy regarding the type, diameter, placement direction, number of screws to be used and number of cortical bone layers to be penetrated, as well as the need and timing of screw removal [16, 20–23]. It is to address these issues that elastic fixation is increasingly replacing the use of cortical screws for DTS fixation, to improve alignment and ankle joint function.

Currently, a button-plate device is the most commonly used elastic fixation method that provides good fixation strength, lowers the risk of complications, and improves postoperative outcomes. Therefore, button-plate elastic fixation aligns with the biomechanical requirements of the ankle joint, allows early postoperative functional exercise, reduces the risk of internal fixation loosening and fracture, and avoids the loss of reduction after removal of screw internal fixation. Overall, elastic fixation reduces pain and the economic burden on patients. In the current review, the AOFAS of suture buttons (91.06) was higher than that obtained with conventional screws (87.78). A prospective study by Degroot et al. [24], comparing cortical screws to suture buttons, reported a comparable rate of poor reduction for both fixation types after surgery, although the rate of poor reduction increased significantly over the 2-year follow-up for cortical screws. Clanton et al. [25] conducted a biomechanical analysis on cadavers, to compare cortical screws, single-suture-button devices, and double-suture-button devices. Their findings indicated that although all three fixation techniques for the DTS provided significant rotational stability to the ankle joint, none of the techniques completely restored the natural anatomy of the distal tibiofibular joint nor rotational stability and the range of internal and external rotation. Of note, the initial tension and firmness of the suture-button device can vary greatly among different surgeons and the device can cause inflammation at the plate site, inducing osteolysis in the area of the button plate and device sinking [3, 6, 24, 26].

In recent years, new fixation techniques of the distal tibiofibular joint have been introduced. Zhao et al. [27] described the use of an arc-shaped Ni–Ti memory osteotomy to provide monolayer cortical bone fixation of the distal tibiofibular joint, which maintains the distal tibiofibular joint space and preserves function of the DTS and ankle joint, with a low incidence rate of postoperative implant complications, such as fracture and loosening. Che et al. [28] proposed that anatomical reconstruction of the DTS, using the peroneus brevis tendon, is possible, providing a firm fixation while maintaining function. However, the arc-shaped Ni–Ti memory osteotomy and peroneus brevis tendon repair are difficult to perform, requiring a steep learning curve; therefore, the use of these two techniques has been difficult to popularize in primary care hospitals.

In this study, we used a nice knot for fixation of the DTS, a simple technique that does not require special instruments. Hill et al. [12] confirmed the positive biomechanical characteristics of the nice knot. The findings of our study showed that use of the nice knot provided a high-strength elastic fixation of the DTS, with no complications, such as loss of reduction or fixation failure, over the follow-up period. In addition, time to partial and to complete weight bearing was significantly earlier in the EB than that in the CS group, with the AOFAS at postoperative 3 months being significantly higher for the EB than that for the CS group. The advantages of the nice knot to bind the tibiofibular syndesmosis can be summarized as follows. First, the nice knot is a double-line sliding knot, with a very high tension achieved by gradually pulling the tail side of the thread while continuously sliding and pressing the knot. After locking, using 3–4 single knots, the knot cannot easily loosen and, thus, the high tension and stability of the fixation are maintained. Second, as an elastic fixation technique, the nice knot aligns more closely with the biomechanical characteristics of the distal tibiofibular joint. Moreover, because of the elastic nature of the fixation, despite a poor reduction of the distal tibiofibular joint during surgery, the distal tibiofibular space can be gradually restored with ankle motion. Third, the encircling and binding technique carries no risk of screw fracture, does not require a second surgery for removal, reduces the economic burden on patients, and is more easily tolerated by older patients. Fourth, elastic stabilization allows early mobilization and return to weight-bearing function, which can facilitate recovery of joint function and reduce the occurrence of long-term complications such as traumatic arthritis. The nice knot has been well designed, with the bone canal selected close to the anterior edge of the fibula and, thus, the knot cannot easily loosen. In addition, compared to the suture-button device and cortical bone screw, the nice knot avoids damage to the fibula and lowers the risk of complications, such as osteolysis, tibia drilling enlargement, and device sinking. Fifth, the nice knot technique is much simpler than the sewing of a button device as it does not require training on the steel wire fixation technique, has a short learning curve, and, thus, can easily be applied in primary care hospitals. Sixth, for high-impact trauma leading to DTS injury often combined with fractures of the fibula, the requirement of lateral plates for fixation of fibular fractures hinders placement of the distal tibiofibular screw and makes needle insertion, 3 mm from the tip of the lateral malleolus, difficult. Lastly, the nice knot technique uses a single bone tunnel and requires pulling only on the tail end to achieve tension and compression of the knot, which is an easy-to-apply surgical technique that decreases the operative time to achieve fixation of the tibiofibular joint.

The limitations of our study need to be acknowledged. First, a biomechanical analysis was not performed and, therefore, we could not confirm that the encircling and binding technique provided ideal mechanical properties of the DTS, such as fixation strength and restraint against rotational stress. Second, radiographs were not performed using a standardized method and, therefore, differences in patient posture may have affected the quality of the radiographs, thus influencing measures of the distal tibiofemoral clear joint space and overlap. Besides, as with all retrospective studies, there are risks of confounding factors that may influence outcome measures between the groups. Lastly, the small sample size was also a limitation of this study. Large sample randomized controlled trials and long-term follow-up are needed to further verify that the method proposed in this study has good clinical effects.

## Conclusions

Elastic fixation, using encircling and binding, compared to cortical screw fixation for DTS injury, provided fixation of sufficient strength, allowing earlier mobilization and time to partial and full weight bearing, thus maintaining the physiological function of the DTS and ankle joint. Moreover, elastic fixation is easier to perform than cortical screw fixation, which, combined with the better operative and clinical outcomes, support its adoption in clinical practice.

## Data Availability

Data and materials supporting the conclusions of this study are available from the corresponding author on reasonable request.

## Abbreviations

AOFAS: American Foot Surgery Association Ankle-Hindfoot Score
CS: Cortical screw
DTS: Distal tibiofibular syndesmosis
EB: Encircling and binding
ROM: Range of motion
TFCS: Tibiofibular space
TFOS: Tibiofibular overlap distance
AS: Visual analog pain score
